# Comparative metabolomics analysis of amphotericin B high-yield mechanism for metabolic engineering

**DOI:** 10.1186/s12934-021-01552-z

**Published:** 2021-03-09

**Authors:** Bo Zhang, Yu Chen, Sheng-Xian Jiang, Xue Cai, Kai Huang, Zhi-Qiang Liu, Yu-Guo Zheng

**Affiliations:** 1 Department, Key Laboratory of Bioorganic Synthesis of Zhejiang Province, College of Biotechnology and Bioengineering, Zhejiang University of Technology, Hangzhou, 310014 People’s Republic of China; 2 Engineering Research Center of Bioconversion and Bio-Purification, Ministry of Education, Zhejiang University of Technology, Hangzhou, 310014 People’s Republic of China

**Keywords:** Amphotericin B, *Streptomyces nodosus*, Comparative metabolomics analysis, Rational guidance, Gene overexpression

## Abstract

**Background:**

The polyene macrocyclic compound amphotericin B (AmB) is an important antifungal antibiotic for the clinical treatment of invasive fungal infections. To rationally guide the improvement of AmB production in the main producing strain *Streptomyces nodosus*, comparative metabolomics analysis was performed to investigate the intracellular metabolic changes in wild-type *S. nodosus* ZJB20140315 with low-yield AmB production and mutant *S. nodosus* ZJB2016050 with high-yield AmB production, the latter of which reached industrial criteria on a pilot scale.

**Results:**

To investigate the relationship of intracellular metabolites, 7758 metabolites were identified in mutant *S. nodosus* and wildtype *S. nodosus* via LC–MS. Through analysis of metabolism, the level of 26 key metabolites that involved in carbon metabolism, fatty acids metabolism, amino acids metabolism, purine metabolism, folate biosynthesis and one carbon pool by folate were much higher in mutant *S. nodosus*. The enrichment of relevant metabolic pathways by gene overexpression strategy confirmed that one carbon pool by folate was the key metabolic pathway. Meanwhile, a recombinant strain with gene *metH* (methionine synthase) overexpressed showed 5.03 g/L AmB production within 120 h fermentation, which is 26.4% higher than that of the mutant strain.

**Conclusions:**

These results demonstrated that comparative metabolomics analysis was an effective approach for the improvement of AmB production and could be applied for other industrially or clinically important compounds as well.

**Supplementary Information:**

The online version contains supplementary material available at 10.1186/s12934-021-01552-z.

## Highlight

1. Comparative metabolomics analysis was applied on wild-type *Streptomyces nodosus* and its mutant to investigate the mechanism of amphotericin B overproduction for the first time.
2. One carbon pool by folate was confirmed as the key metabolic pathway related to amphotericin B biosynthesis.
3. The overexpression of genes *MetH, GlyA* and *PurN* in high AmB-yield strain all exerted positive impacts on AmB accumulation in comparison with the control.

## Background

Amphotericin B (AmB) is an important macrocyclic polyketide mainly produced by *Streptomyces nodosus*, it has various biological and pharmacological activities, such as broad-spectrum antifungal [1], antiparasitic [2, 3], antitumor and anti-HIV activities [4]. The fungal infections, especially invasive fungal infections, are expanding in recent years and becoming more stubborn to be cured [5]. AmB plays a crucial role in inhibiting the growth of fungi by affecting the permeability of cell membranes and thus was used to effectively treat invasive fungal infections. In order to reduce the toxic and side effects of AmB, some strategies have been developed, including water-soluble dextrin-AmB (Dex-AmB) formulations and AmB loaded pluronic F127 (PF 127) micelles which was then coated with chitosan (Cs-PF-AmB-M) [6, 7].

As AmB is a complex compound, the synthesis of AmB by chemical way is extremely difficult. In contrast, the biosynthesis of AmB by microorganism is relatively convenient and environmental-friendly. The gene cluster for AmB biosynthesis belongs to the type I polyketide synthase (PKS), the process of which including three steps: (i) the biosynthesis of precursors, i.e. three propionate and 16 acetate precursors, (ii) the extension of the PKS chain and (iii) the modification of post-PKS. The precursors are cyclized under the action of PKS to form the macrolactone core. On this basis, the target product AmB is then obtained by the oxidation of a methyl branch at C_41_, mycosaminylation at C_19_ [8] and hydroxylation at C_8_ sequentially. The biosynthesis of these precursors involved in multiple primary metabolic pathways, such as central carbon metabolism, fatty acid metabolism and amino acid metabolism, etc. The variations of intracellular metabolite in organism related to the above pathways are essential to study the potential key factors for AmB biosynthesis. Recently, metabolomics-based approach has been applied as a powerful tool to measure and evaluate the dynamic multiparametric metabolic responses of living systems to the inside and outside disturbance in a comprehensive manner. For example, comparative metabolomics approach was employed to analyze metabolite concentration differences of *Streptomyces tsukubaensis* cultivated in two media [9]. Metabolomics results illuminated that oxygen, *S*-adenosine methionine (SAM) and the transport protein were vital limiting factors for AmB accumulation in *S. nodosus* [10]. Meanwhile, through optimization of fermentation process, changes of α-ketoglutarate, pyruvate and citric acid concentration were identified as the most critical metabolite nodes for AmB biosynthesis [11]. Based on these results, further comparative metabolomics analysis can be useful for metabolic engineering of strain for AmB overproduction. Metabolic engineering has been applied based on analysis of metabolic network in *Escherichia coli*, *Streptomyces* and *Corynebacterium glutamicum* for the industrial production of valuable amino acids and chemicals, such as L-2-aminobutyric acid, shikimic acid, ascomycin and so on [12–14].

In this work, comparative metabolomics analysis was employed to analyze the intracellular metabolite variations between mutant and the wild-type *S. nodosus*. The potential key metabolites that might be responsible for AmB overproduction were identified for the first time. Afterwards, the variations of these metabolite were evaluated and analyzed to reveal the possible factors that influencing the AmB biosynthesis. Because the changes of metabolites reflected the changes of metabolic pathway fluxes, gene overexpression strategy at key metabolic nodes was carried out to further improve AmB production based on the above analysis.

## Results

### Differences of fermentation kinetics between the wild-type and mutant *S. nodosus*

**Fig. 1:**
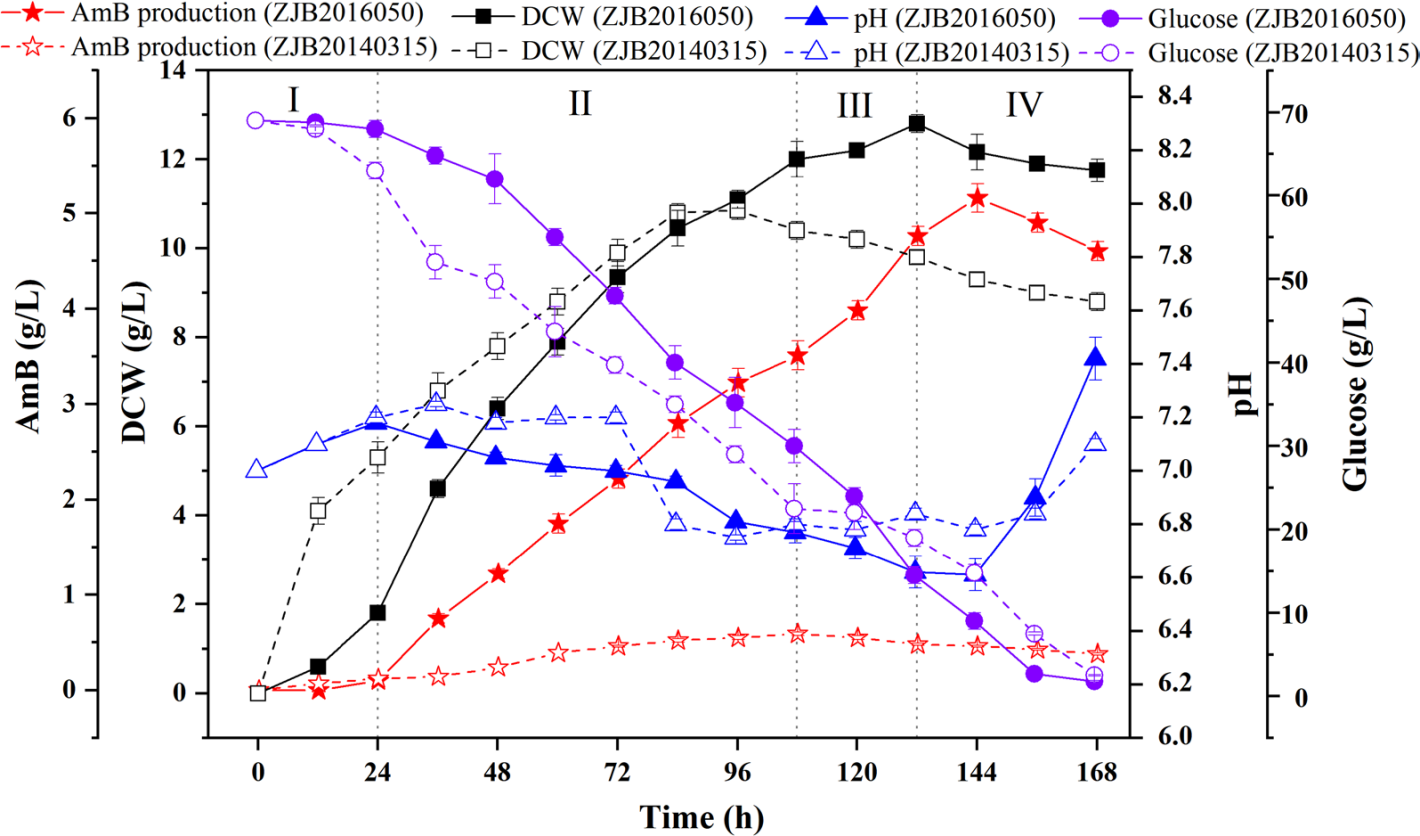
Time course profiles of key fermentation parameters in mutant *S.nodosus* ZJB2016050 and wild-type *S.nodosus* ZJB20140315. Four profiles were illustrated in the line chart, including the yield of AmB, dry cell weight, pH and residual glucose. The whole process could be divided into four phases, lag phase (0–24 h), exponential phase (24–108 h), stationary phase (108–132 h) and decline phase (132–168 h). Error bars show standard derivation among three experiments

To find out the internal changes of mutant *S. nodosus*, fermentation kinetics for AmB production was studied. Wild-type and mutant strains under the same cultivation condition showed the same fermentation cycle which could be divided into lag phase (phase I, 0–24 h), logarithmic phase (phase II, 24–108 h), stationary phase (phase III, 108–132 h) and decline phase (phase IV, 132–168 h) (Fig. 1). In the lag phase, pH values in the medium showed no significant differences, but AmB could be detected in mutant *S. nodosus* at 24 h while no AmB was detected in wild-type *S. nodosus*. In addition, the biomass increased rate and total sugar consumption rate of wild-type *S. nodosus* were faster than that of mutant *S. nodosus*. The average sugar consumption rate between two strains showed significant discrepancy between 24 and 48 h, which indicate the metabolism distinction between two strains.

In the logarithmic phase, the biomasses of two strains increased rapidly and the pH values declined. Meanwhile, the average sugar consumption rate between 60 and 108 h remained stable. Additionally, the production of AmB at 72 h reached about 0.5 g/L in wild-type *S. nodosus* and 2.8 g/L in mutant *S. nodosus*, respectively, and the difference of AmB production in two strains began to be noticeable (> 2 g/L) at this time.

In the stationary phase, the biomasses and the pH values between two strains were obviously different, especially for the average sugar consumption rate, which increased rapidly in mutant *S. nodosus*. To be noticed, a large percent of AmB was accumulated in this phase, indicating that this phase was the principal phase for AmB production in wild-type and mutant strains. In the decline phase, the yield of AmB began to decrease and the pH increased significantly in wild-type and mutant *S. nodosus*.

Taken together, the fermentation advantages of mutant *S. nodosus* for AmB production were obvious, with a 26.4% increase for AmB production. However, the potential factors that affect AmB production were obscure and could not be known through the analysis of fermentation kinetics. It was necessary to fully study the changes of intracellular metabolites as well as the strain metabolism discriminations between the wild-type and the mutant strain.

### Comparative metabolomics analysis

**Fig. 2:**
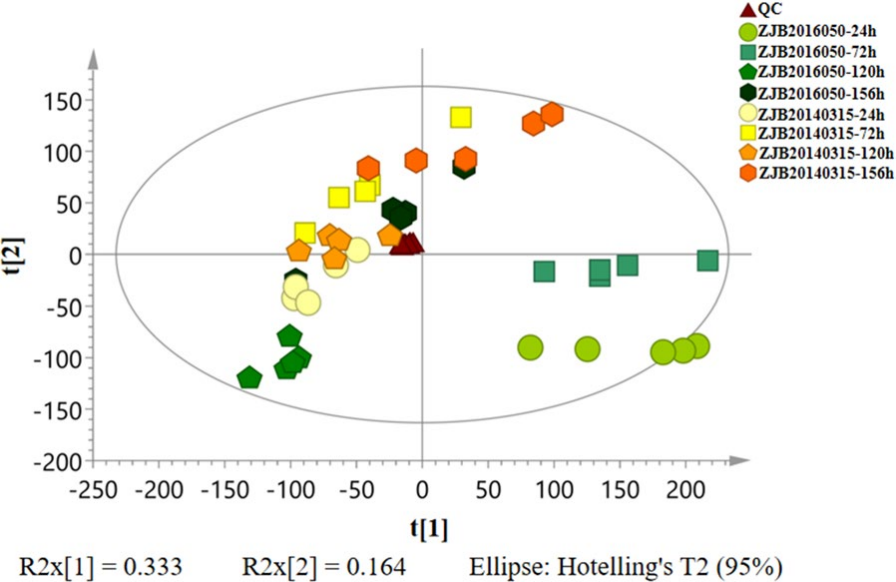
PCA analysis of intracellular metabolites at different fermentation time points. The Principal Component Analysis model with R2x [1] of 0.333, R2x [2] of 0.164 based on the metabolic analysis and AmB production. In order to assess the accuracy and stability of the equipment state during detection and collection process, the QC samples were prepared in advance and tested every 10 samples

**Fig. 3:**
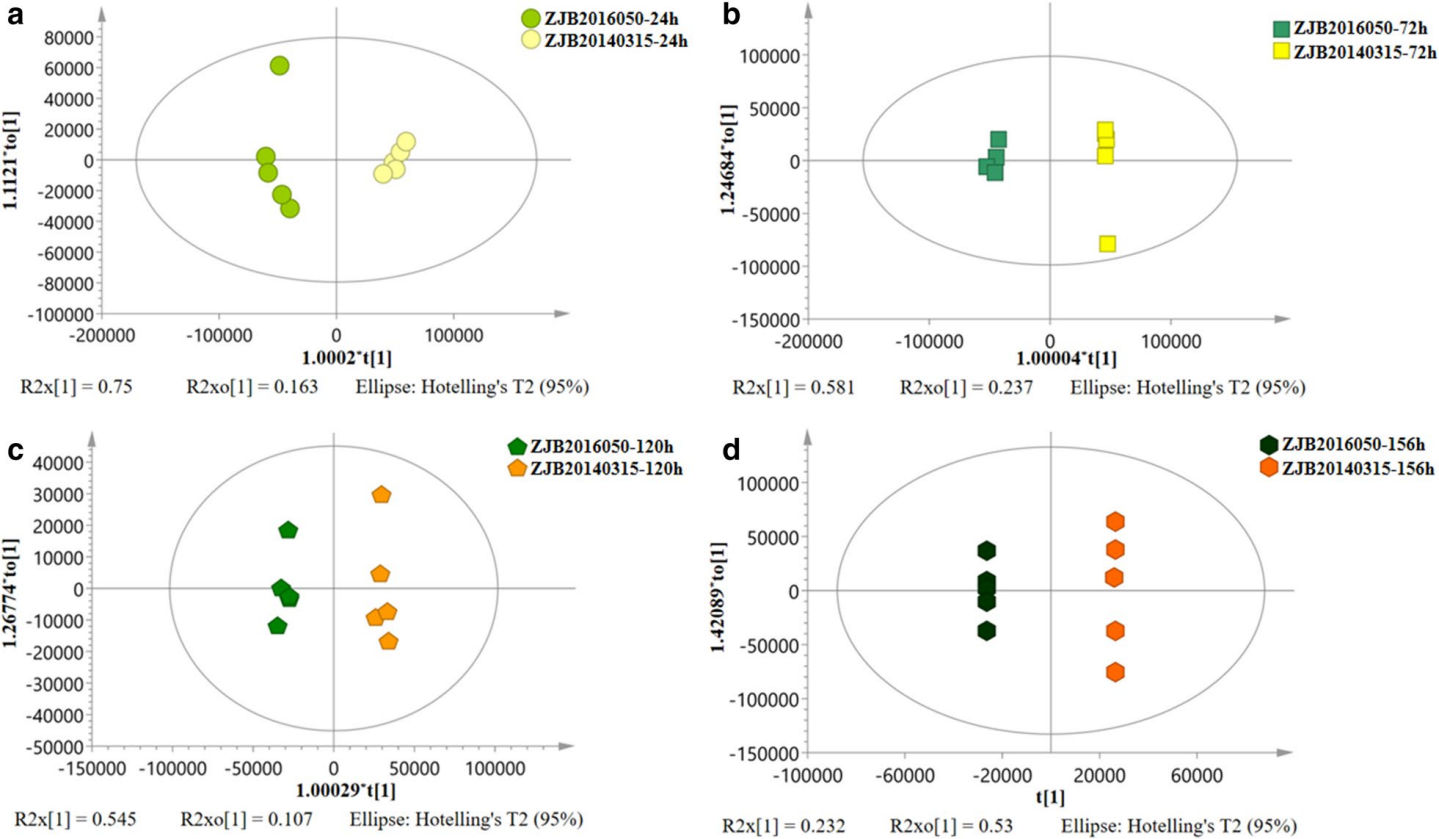
The Orthogonal Partial Least Squares-discriminate Analysis model based on the metabolic analysis and AmB production. **a**–**d** The Orthogonal Partial Least Squares-discriminate Analysis of mutant *S. nodosus* and wild-type *S. nodosus* at 24 h, 72 h, 120 h and 156 h

To investigate the discrepancies of intracellular metabolites between wild-type and mutant strain, the raw data of LC–MS were imported into Progenesis QI software, results indicated that 7758 compounds were identified. Here, PCA analysis examined the stability of the process and the overall distribution between samples (Fig. 2). (O)PLS-DA distinguished more clearly for the sample groups and could improve the model effectiveness (Fig. 3). Concretely, in order to assess the accuracy and stability of the equipment state during detection and collection process, the points of the quality control (QC) samples were closely clustered together in the PCA score plot. Most samples within 95% confidence intervals were close to each other and the sample repeatability qualities were reliable. The differences were small when the sample marks gathered, whereas the differences were great when the sample marks dispersed.

**Fig. 4:**
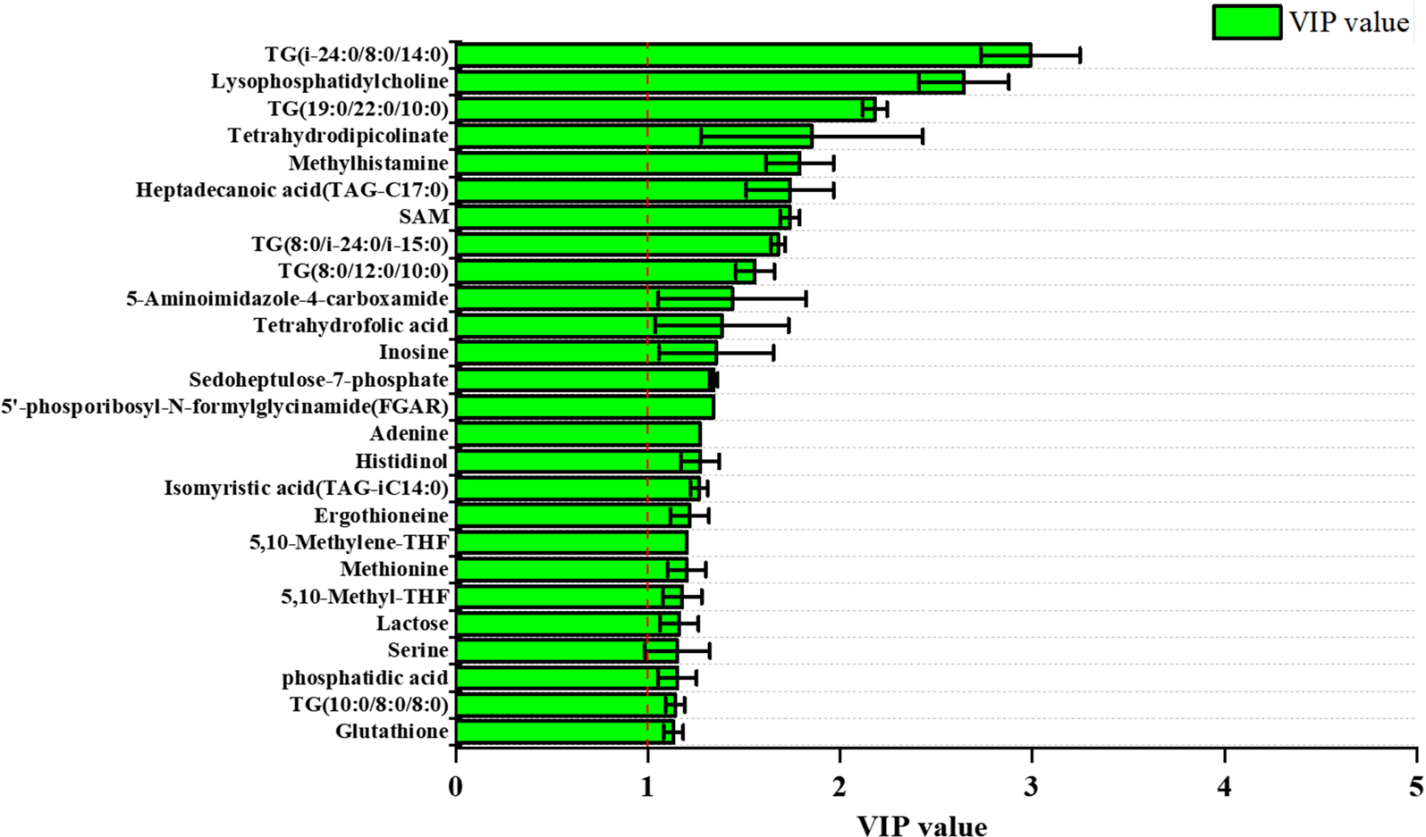
The VIP plot of the (O)PLS-DA model generated from the intracellular metabolites. The VIP values represented the contribution of metabolites, and the higher the VIP value of a metabolite, the more contribution that this metabolite showed. The variables with VIP value greater than 1 are separated from the others by a red line

In the (O)PLS-DA score plot, both groups of samples marks were distributed on different sides of the Y axis in each fermentation stage, and the distinction was significant. The results showed that (O)PLS-DA method analysis could reduce the difference between the same group of samples and make the difference between different groups of samples more significant. The significant discrimination on the (O)PLS-DA score plot profiling revealed the metabolic discrimination of the wild-type strain and its mutant strain. The variable importance in the projection plot demonstrated the contribution of each identified metabolite to sample class separation and AmB accumulation. Generally, a metabolite with a VIP value greater than 1 indicated a significant contribution to the separation of sample groups within (O)PLS-DA model (Fig. 4). The higher the VIP value of a metabolite, the higher contribution of this metabolite was shown for the AmB accumulation [15]. The identified metabolites with VIP values greater than 1 were listed in Additional file 1: Table S3, and most of them were positively correlated to the AmB yield. These metabolites (VIP > 1) were mainly involved in carbon metabolism, fatty acid metabolism, amino acid metabolism, purine metabolism, folate biosynthesis and one carbon pool by folate. To explore the key factors involving in the AmB production, the key metabolites with VIP values greater than 1 were comparatively analyzed.

**Fig. 5:**
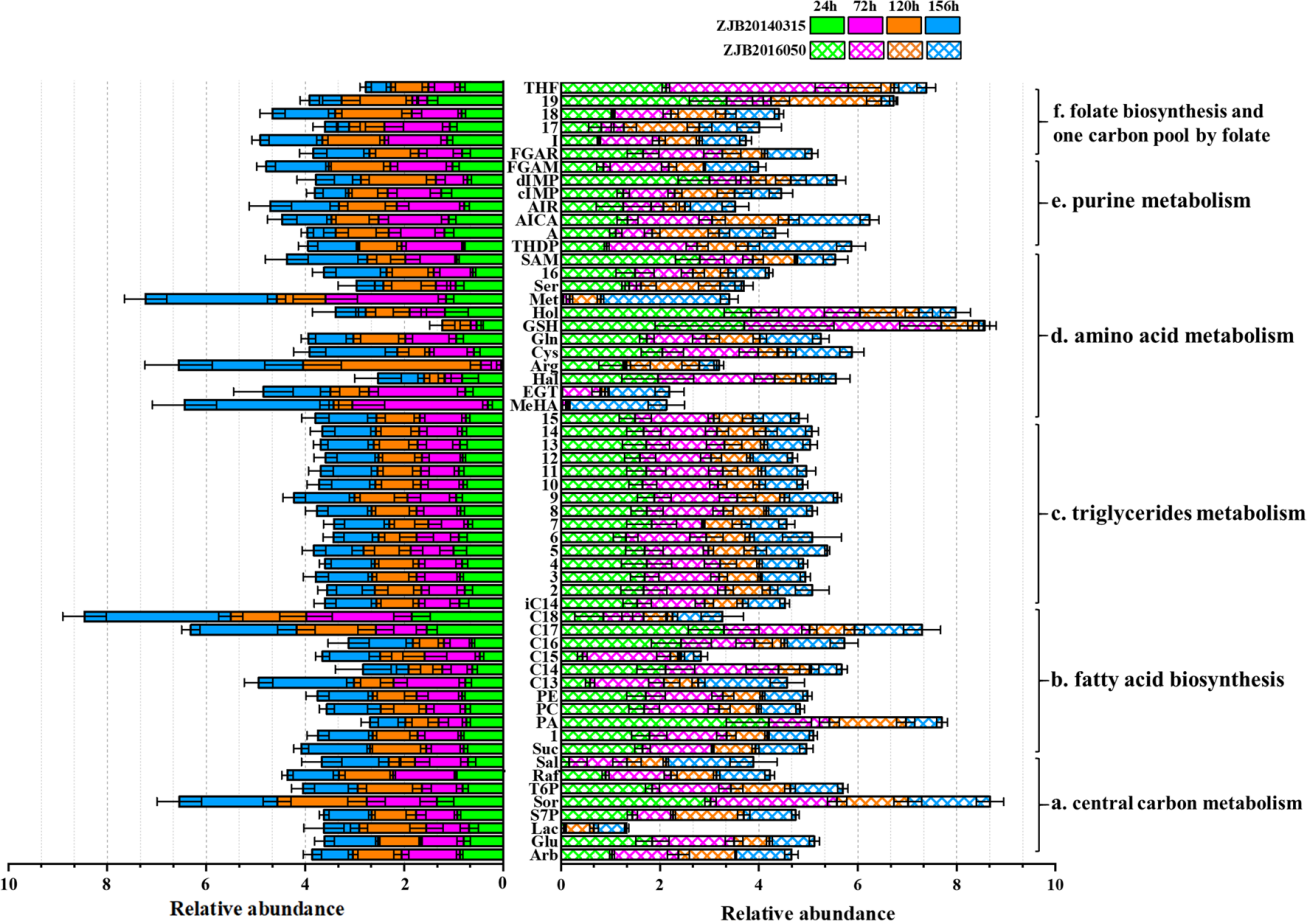
The relative abundance of various intracellular metabolites in different fermentation period. Metabolites were analyzed to investigate the differences during the whole fermentation process of *S. nodosus*, including metabolism of central carbon, fatty acids, triglycerides, amino acids, purine, folate biosynthesis and one carbon pool by folate. **a** Central carbon metabolism. *Arb* arbutin, *Glu* glucose, *Lac* lactate, *S7P* sedoheptulose-7-phosphate, *Sor* sorbitol, *T6P* tagatose-6-phosphate, *Raf* raffinose, *Sal* salicin, *Suc* succinic acid. **b** Fatty acid biosynthesis. *1* lysophosphatidylcholine, *PA* phosphatidic acid, *PC* phosphatidyl choline, *PE* phosphatidyl ethanolamine, *C13* tridecanoic acid, *C14* myristic acid, *C15* pentadecanoic acid, *C16* palmitic acid, *C17* heptadecanoic acid, *C18* stearic acid, *iC14* isomyristic acid. **c** Triglycerides metabolism. 2, TG(10:0/10:0/8:0); 3, TG(10:0/8:0/8:0); 4, TG(10:0/i-20:0/14:0); 5, TG(12:0/12:0/12:0); 6, TG(14:0/18:0/16:0); 7, TG(16:0/15:0/18:0); 8, TG(19:0/22:0/10:0); 9, TG(20:0/i-12:0/i-18:0); 10, TG(8:0/12:0/10:0); 11, TG(8:0/8:0/8:0); 12, TG(8:0/i-24:0/i-15:0); 13, TG(i-20:0/i-20:0/8:0); 14, TG(i-24:0/8:0/14:0); 15, TG(i-24:0/i-13:0/10:0). **d** Amino acid metabolism. *MeHA* methylhistamine, *EGT* ergothioneine, *Hal* histidinal, *Arg* arginine, *Cys* cysteine, *Gln* Glutamine, *GSH* glutathione, *Hol* histidinol, *Met* methionine, *Ser* serine, *16* Phosphoribosyl-AMP, *SAM* *S*-Adenosylmethionine, *THDP* tetrahydrodipicolinate. **e** Purine metabolism. *A* adenine, *AICA* 5-aminoimidazole-4-carboxamide, *AIR* 5-aminoimidazole ribonucleotide, *dIMP* deoxyinosine monophosphate, *FGAM* formyl glycinamidine ribonucleotide, *FGAR* formyl-glycinamide ribonucleotide, *I* inosine. **f** Folate biosynthesis and one carbon pool by folate. 17, 5,10-methylenetetrahydrofolic acid; 18, 5-formiminotetrahydrofolic acid; 19, 5-methyltetrahydrofolic acid; THF, tetrahydrofolic acid. The X-axis showed the relative abundance of metabolites. The Y-axis showed the different metabolites. Each value shown represented the mean of five independent experiments and the error bars represented standard deviations of five values

Significant discrepant metabolites were identified among four groups with VIP > 1 and *p* value < 0.05. Results indicated that 674 differential metabolites between mutant *S. nodosus* and wild-type *S. nodosus* were identified at 24 h, 613 differential metabolites at 72 h, 721 differential metabolites at 120 h, and 386 differential metabolites at 156 h. In this work, 60 differential metabolites were determined to display a marked difference between two strains according to the statistical analysis of differential metabolites at 24 h, 72 h, 120 h and 156 h of fermentation process (Fig. 5). Metabolite data were analyzed to study the differences during the whole fermentation process of *S. nodosus*, including carbon metabolism, fatty acid metabolism, amino acid metabolism, purine metabolism, folate biosynthesis and one carbon pool by folate.

### Comparative metabolites in central carbon metabolism analysis

The results of (O)PLS-DA analysis indicated that glucose, lactate, tagatose-6-phosphate, sorbitol, sedoheptulose-7-phosphate and succinate had greater contribution (VIP > 1) to the AmB accumulation. The changes of intracellular metabolites involved in carbon metabolism were shown in Fig. 5a. Compared to wild-type *S. nodosus*, the levels of glucose, tagatose-6-phosphate, sorbitol and intermediate metabolites sedoheptulose-7-phosphate were higher in mutant *S. nodosus*, and they all decreased during the main period of amphotericin biosynthesis (24–120 h) (Fig. 5a). This indicated that mutant *S. nodosus* could utilize sugar better than the wild-type strain. However, the levels of lactate were very little in mutant *S. nodosus*, and it showed a decreasing trend in wild-type *S. nodosus* during the logarithmic period (24–72 h). One possible explanation for above observations was that lactate was a competitive branch of pyruvate metabolism flowing to propionic acid and acetyl-CoA. Reducing the accumulation of lactate would result to increased acetyl-CoA pool which was then entered into the TCA cycle.

### Comparative metabolites in fatty acid metabolism analysis

Compared to wild-type *S. nodosus*, the levels of major fatty acids and phospholipids were higher in mutant *S. nodosus*, and they all decreased during the main period of amphotericin biosynthesis (24–120 h) (Fig. 5b). It has been reported that under aerobic conditions, metabolism of fatty acids can decompose and release a large amount of energy and acetyl-CoA, which can be carboxylated to form malonyl-CoA. To be noticed, acetyl-CoA, malonyl-CoA and methylmalonyl-CoA were important precursors for the synthesis of AmB.

Hexadecanoic acid, heptadecanoic acid, myristic acid, pentadecanoic acid, phosphatidic acid and triglycerides (TGs) (Fig. 5c) were the key metabolites that affected metabolism and the production of AmB according to (O)PLS-DA analysis. Phosphatidic acid is a component of cell membrane as a prerequisite for the synthesis of phosphoglycerides, and its corresponding phosphate could be combined with some molecules to form a variety of phospholipids.

TGs are lipids formed by glycerol and fatty acids, and it is reported that triacylglycerol (TAGs) plays a key role in linking primary metabolism to polyketide synthesis in *streptomyces* [16]. Intracellular TAGs, which were accumulated in primary metabolism, could be degraded during stationary phase in mutant *S. nodosus*. This process could channel carbon flux from both intracellular TAGs and extracellular substrates into polyketide biosynthesis pathway. Compared with mutant *S. nodosus*, concentrations of TGs in wild-type *S. nodosus* were almost unchanged. It showed that the synthesis of polyketide was weak in wild-type *S. nodosus*.

Therefore, it could be speculated that the fatty acids could regulate cell metabolism from primary to secondary metabolism and then affected the synthesis of AmB polyketide.

### Comparative metabolites in amino acid metabolism analysis

The results of (O)PLS-DA analysis indicated that methionine, cysteine, SAM, arginine, tetrahydrodipicolinate and glutathione were the key metabolites that affected cell metabolism and the AmB biosynthesis (Fig. 5d). SAM is an important molecule to maintain normal cell function and survival, involved in three important metabolic pathways: transmethylation, transmercaptylation and polyamine synthesis [17]. A number of literatures have been reported that gene *metK* (coding SAM synthetase) can improve the synthesis of SAM and increase the production of secondary metabolites in *Streptomyces* [18, 19]. Compared to wild-type *S. nodosus*, the levels of methionine supply were especially insufficient in mutant *S. nodosus*. Methionine is a precursor to SAM, and gene *metH* (methionine synthase) has been reported to improve methionine synthesis [20].

Similarly, compared to wild-type *S. nodosus*, the levels of glutathione (GSH) were higher in mutant *S. nodosus*, and they all decreased during the main period of amphotericin biosynthesis (24–120 h). It is reported that GSH maintains bacteria homeostasis through enzymatic reactions and participates in metabolic redox reactions. In addition, GSH plays an important role in cell resistance to high oxygen, high osmotic pressure, metal ions and environmental changes during the fermentation process.

Results also showed that the level of arginine decreased a lot in the fermentation early stage of mutant *S. nodosus*, and arginine could be metabolized to spermine containing two amino groups and two imino groups. Spermine can make DNA molecules more flexible and stable in bacteria and is deemed as an important substance to promote cell proliferation. Moreover, a high level of tetrahydrodipicolinate was measured in mutant *S. nodosus*, while tetrahydrodipicolinate is involved the formation of racemic 2,6-diaminyl heptadiic acid, which can be used in the biosynthesis of lysine and also be used as one of the bacterial peptidoglycan components to form the cell wall structure [21]. These results indicated that changes of amino acid metabolism in mutant *S. nodosus* would help the strain for growth and thus promote the biosynthesis of AmB.

### Comparative metabolites in purine metabolism analysis

Nucleotides are mainly involved in the formation of nucleic acids, and many single nucleotides also involved in important biological functions. Compared to wild-type *S. nodosus*, the levels of purine metabolic related chemicals showed more obvious decreasing trend in mutant *S. nodosus*, such as glutamine, 5′-phosphoribosyl-*N*-formylglycinamide (FGAR) and 5-aminoimidazole ribonucleotide (AIR) (Fig. 5e). Additionally, there was little difference for the 5-aminoimidazole-4-carboxamide (AICA) between the wild-type *S. nodosus* and mutant *S. nodosus*.

### Comparative metabolites in folate biosynthesis and one carbon pool by folate analysis

The results of (O)PLS-DA analysis indicated that tetrahydrofolic acid, methyl-THF and methylene-THF showed significant differences between wild-type and mutant strains, and they might be the key metabolites that affected cell metabolism and the AmB production (Fig. 5f). Compared to wild-type *S. nodosus*, the levels of tetrahydrofolic acid and methyl-THF were higher in mutant *S. nodosus*, and the level of methyl-THF decreased during 24 to 72 h, while the level of tetrahydrofolic acid decreased during 72 to 120 h. Almost all the synthesis of nucleic acids, amino acids and pantothenic acids in bacteria required tetrahydrofolic acid as a carrier of carbon units. Combined with the results of methionine metabolism and purine metabolism, it might be concluded that tetrahydrofolic acid was the key metabolite in the mutant *S. nodosus*. The role of tetrahydrofolate in the one carbon pool by folate is based on the conversion between methyl-THF and methylene-THF, and tetrahydrofolate can be obtained by the dihydrofolate reduction catalyzed by dihydrofolate reductase [22].

### The relationships between intracellular metabolites and amphotericin accumulation

**Fig. 6:**
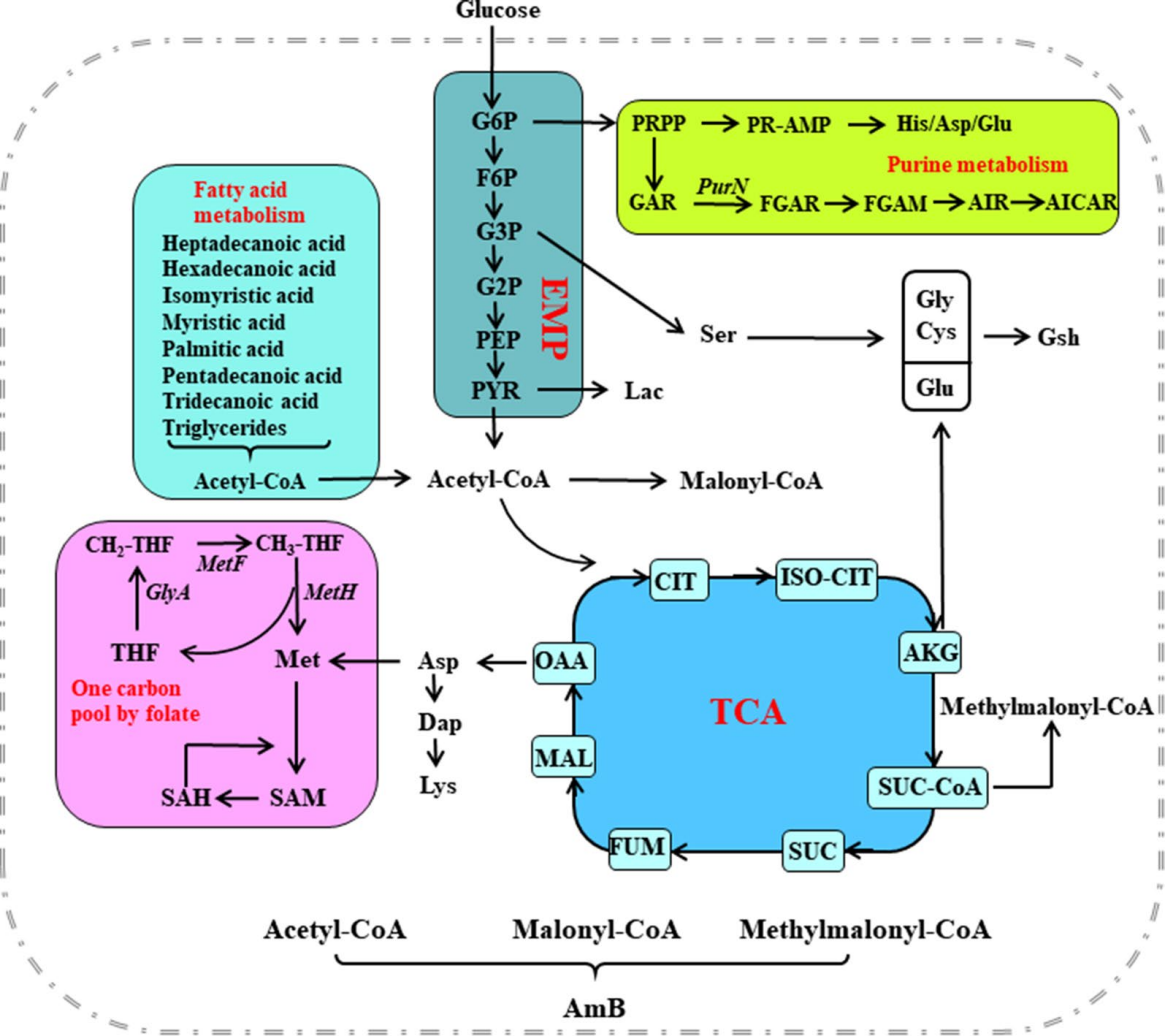
The proposed relationships of amphotericin biosynthesis with relevant metabolic pathways in *S. nodosus.* The key metabolic pathways were marked by red, including central carbon metabolism, fatty acids metabolism, purine metabolism and one carbon pool by folate. *EMP* Embden–Meyerhof–Parnas pathway, *TCA* tricarboxylic acid cycle, *Glu* glucose, *G6P* glucose 6-phosphate, *F6P* fructose 6-phosphate, *G3P* glucose 3-phosphate, *G2P* glucose 2-phosphate, *PEP* phosphoenolpyruvate, *Pyr* pyruvate, *CIT* citrate, *ISO-CIT* iso-citrate, *AKG* α-ketoglutarate, *SUC-CoA* succiny-CoA, *SUC* succinate, *FUM* fumaric acid, *MAL* malic acid, *OAA* oxalacetic acid, *Lac* lactate, *Ser* serine, *Gly* glycine, *Cys* cysteine, *Glu* glutamic, *Gsh* glutathione, *Asp* aspartic, *Dap* diaminopimelate, *Lys* lysine, *His* histidine, *THF* tetrahydrofolic, *Met* methionine, *SAM* *S*-adenosine methionine, *SAH* *S*-adenosyl-l-homocysteine, *PRPP* 5-Phospho-alpha-d-ribose-1-diphosphate, *PR-AMP* phosphoribosyl-adenosine monophosphate, *GAR* glycinamide ribonucleotide, *FGAR* formyl-glycinamide ribonucleotide, *FGAM* formyl glycinamidine ribonucleotide, *AIR* 5-aminoimidazole ribonucleotide, *AICAR* 5-aminoimidazole-4-carboxamide ribonucleotide

To find out the important metabolic pathways for AmB biosynthesis, pathway enrichment analysis and metabolite addition strategies were further employed. The differential metabolites were then analyzed through pathway enrichment based on KEGG database (see Additional file 1: Figures S1 and S2) and the effects of different addition strategies on the production of AmA and AmB were studied, finally, 26 intracellular metabolites were chosen (as shown in Additional file 1: Table S3). Compared to wild-type *S. nodosus*, the levels of these 26 key metabolites were higher in mutant *S. nodosus*, and almost all of them showed decreasing trends during the main fermentation period for amphotericin biosynthesis (24–120 h). These metabolites involved in carbon metabolism, fatty acid metabolism, amino acid metabolism, purine metabolism, folate biosynthesis and one carbon pool by folate, etc. The relationships between these metabolism pathways and amphotericin biosynthesis were revealed and summarized in Fig. 6.

### Gene overexpression strategies for AmB improvement

In this study, mutant strain was obtained by UV-NTG mutagenesis of wild-type *S. nodosus*. The mutations in the genome caused by UV-NTG treatment resulted to the metabolite change in mutant strain. In brief, the changes of metabolite were due to the changes of metabolic flux, which were controlled by gene expression. Hence, we firstly employed the comparative metabolism analysis between mutant and wild-type *S. nodosus* to find out the potential key metabolites. To further improve the AmB production, strategies including addition of the critical metabolites or overexpression of the key genes in the pathway were employed, in which the latter would be more efficient.

**Fig. 7:**
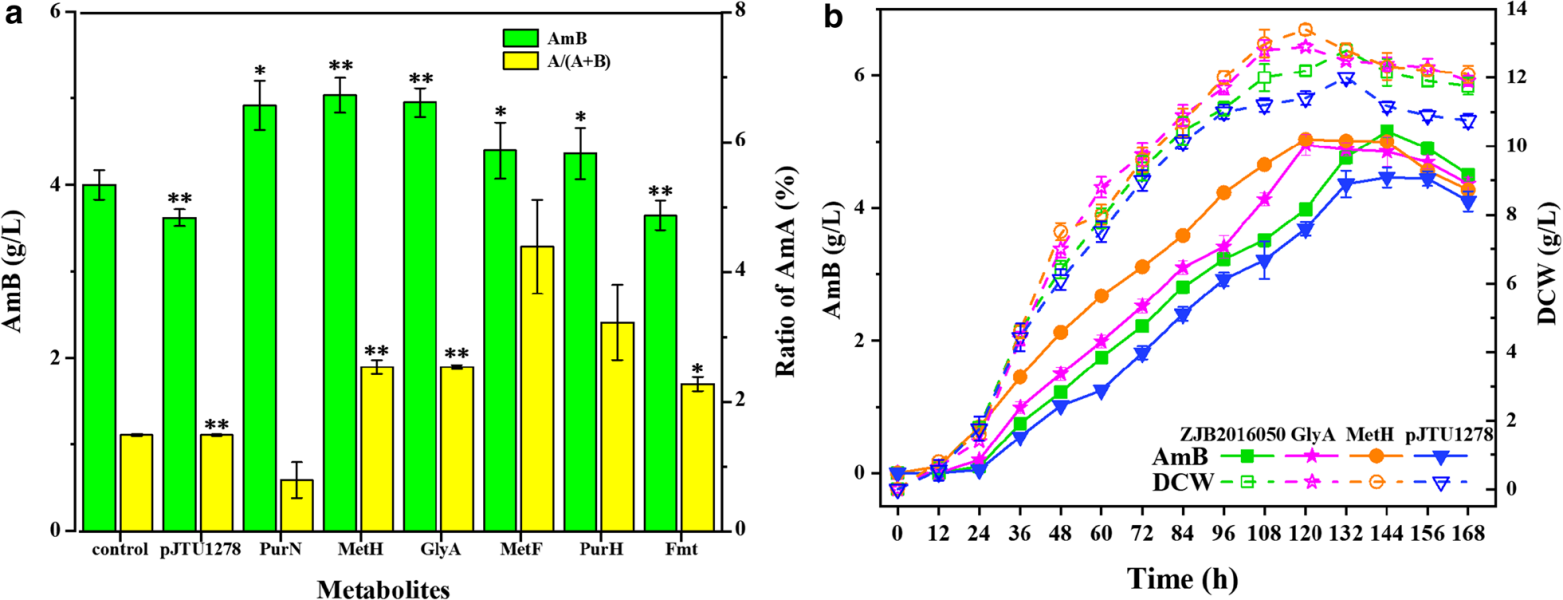
AmB production associated with genes overexpression and the fermentation time courses. **a** AmB production associated with genes overexpression in various engineered strains, the genetically engineered strains were all constructed from primitive strain, mutant *S. nodosus*. Control represented mutant *S. nodosus* strain, pJTU1278 represented mutant *S. nodosus* with plasmid pJTU1278. PurN, metH, glyA, metF, purH and fmt represent overexpression of gene *purN*, *metH*, *glyA*, *metF*, *purH* and *fmt*. Samples were collected from soluble fermentation at 120 h, and the AmB concentration and ration of AmA were detected and analyzed, respectively. Symbol ‘*’ indicates the experimental strain compare with the mutant strain. More p-value details about symbols are * indicates p < 0.05, ** indicates p < 0.01. **b** Fermentation time course for mutant *S. nodosus*, pJTU1278, GlyA and MetH. Mutant *S.nodosus* and pJTU1278 were primitive strain and the strain with empty vector, respectively. MetH and glyA represented overexpression of gene *metH* and *glyA*. Error bars showed standard derivation among three experiments

We had studied the obvious different intracellular tetrahydrofolate levels between wild-type and mutant strains, inadequate supply of methionine and continuously decreased *S*-adenosylmethionine level in mutant *S. nodosus*, which helped to identify that one carbon pool by folate metabolic pathway might be the key metabolic pathway. To verify these results, 50 mg/L methionine, serine, glycine, SAM and THF were selected to study their effect on AmB production in mutant *S. nodosus*. As shown in Additional file 1: Figure S3, methionine, serine, glycine, SAM and THF showed positive affections for AmB biosynthesis which had been reported by our group [10]. Besides this, several gene expression vectors had been constructed (*gcvT*, *thyX*, *fmt*, *metF*, *purH*, *metH*, *purN*, *DHFR* and *glyA*), these genes were overexpressed under the control of promoter ermE*p from *Saccharopolyspora erythraea*. The expression vectors were introduced into the mutant *S. nodosus* by optimized conjugative transferred method and the engineered strains were successfully obtained (Fig. 7a). Strain that overexpressed *metH* showed a 5.03 g/L AmB yield at 120 h in flask shake fermentation, about 26.4% higher than that of the mutant strain *S. nodosus*. RT-qPCR analysis showed the increased transcriptional levels for *purN*, *metH*, *glyA*, *metF*, *purH*, and *fmt* genes in the engineered strains (Additional file 1: Figure S4).

## Discussion

In previous work [23], we obtained a mutant strain named *S. nodosus* ZJB2016050 with high AmB yield by environmental/chemical mutagen (UV+ *N*-methyl-*N*-nitroso-*N*-nitroguanidine) treatment of wild-type *S. nodosus* ZJB20140315. Then, new strategies with overexpressing some key genes involved in oxygen-taking or precursor-acquiring were carried out in our lab. Based on comprehensive analysis of key metabolites and pathways during different AmB fermentation time in mutant strain, we have identified 28 metabolites as key factors and 6 pathways were closely associated with AmB production and studied to optimize the biosynthesis of AmB [10]. However, the results of genes overexpression did not support metabolome analysis results strongly, which indicated the complexity of the metabolic network. Meanwhile, the metabolome analysis results of a single strain were not enough as they only provided the metabolite changes at different fermentation times. What’s more, the differences of the two strains at the same fermentation time were obtained more intuitively, for example, compared with wild-type *S. nodosus*, the level of substances related to fatty acid metabolism especially the TG metabolism decreased obviously in mutant *S. nodosus*. This result indicated that oxygen supply was a crucial factor for providing sufficient precursors for AmB biosynthesis. In addition, some results showed that SAM precursor-methionine was inadequately supplied and we found obvious different tetrahydrofolate levels between the mutant and wild-type strain, which could not be obtained from previous metabolome analysis. Hence, the comparative metabolome analysis between mutant *S. nodosus* and wild-type *S. nodosus* was necessary to obtain the potential clues for the high-production of AmB by mutant strain.

In this study, in order to rationally guide the improvement of AmB production, the discrepancy between intracellular metabolism and AmB accumulation in mutant and wild-type *S. nodosus* were studied by comparative metabolomics analysis. The results of (O)PLS-DA analysis indicated that there were strong correlations between intracellular metabolites and AmB accumulation.

In carbon metabolism, the decrease of lactate levels could improve acetyl-CoA pool which was then entered to the TCA cycle. As an important intermediate metabolite of primary metabolites, sedoheptulose-7-phosphate was also considered as a node for AmB production. In fatty acid metabolism, most of the fatty acids could be catabolized to release large amount of energy and acetyl-CoA, phosphatidic acid with high level in mutant strain is an important component of the cell membrane and participates in the recognition and signal transduction of membrane proteins. In addition, TGs can regulate cell primary metabolism to secondary metabolism, thereby affecting the synthesis of secondary metabolite AmB. In the study of amino acid metabolism, GSH maintained a relative high level at the fermentation early stage in mutant strain, as GSH could keep the cell intracellular redox balance through enzymatic reactions which helped strain adapt to environmental changes. As a methyl donor of the secondary metabolite, SAM was a key metabolite and was found to be lack at the fermentation early stage for AmB biosynthesis. In the study of purine metabolism, purine and pyrimidine were growth factors, but significant down-regulations of FGAM, AIR and other precursors in the biosynthesis of AICAR were showed in the mutant strain. In the one carbon pool by folate, the levels of tetrahydrofolic acid and methyl-THF were higher in mutant *S. nodosus* than in the wild-type strain. All these results showed the advantages in mutant *S. nodosus* for high AmB production. As most of nucleic acids, amino acids and pantothenic acids in bacteria required tetrahydrofolic acid as a carrier of carbon units, tetrahydrofolic acid was deemed as the key metabolite in the mutant *S. nodosus*.

Moreover, 26 key metabolites that contributed to AmB productivity were identified, most of which were related to carbon metabolism, fatty acid metabolism, amino acid metabolism, purine metabolism and folate biosynthesis. The relationships between these metabolism and amphotericin biosynthesis are summarized in Fig. 6. According to the analyses and results of comparative metabolomics, one carbon pool by folate metabolic pathway was identified as the key metabolic pathway. Based on this, further study of the effects of precursors and key metabolites including serine, glycine and SAM addition on AmB production were carried out in our previous studies [10, 11] and the addition of methionine and THF showed improved AmB yield (Additional file 1: Figure S3). Later, gene overexpression strategies were conducted in mutant *S. nodosus* to improve AmB production according to the above results. We screened the genes that were involved in one carbon pool by folate metabolic pathway. Through overexpression of single gene *purN*, *metH*, *glyA*, *metF*, *purH* or *fmt*, a strain with gene *metH* overexpression was obtained with its optimal AmB yield at 5.03 g/L in 120 h fermentation, which increased by 26.4% compared with that of the mutant strain *S. nodosus* in flask shake fermentation. However, the yield of by-product AmA in those recombinant bacteria was unstable, as AmA decreased in the strain with *purN* overexpressed and obviously increased in the strain with other genes overexpressed. Recently, Huang et al. [24] speculated that the decrease synthesis of AmA was related to the insufficient NADPH or the weak binding force between ER5 domain and NADPH. Based on critical role of ER5 domain of *amphC* in the biosynthesis of AmA and AmB reported by Cafrey [1], reducing the by-product AmA would become the focus of our subsequent studies.

## Conclusions

Overall, based on the comparative metabolome analysis between mutant and wild-type *S. nodosus*, one carbon pool by folate metabolic pathway was identified as the key metabolic pathway in the mutant strain. By choosing the genes related to the one carbon pool by folate metabolic pathway for overexpression, three recombinant strains with the overexpression of key metabolic genes (*purN*, *glyA* and *metH*) in *S. nodosus* were obtained and they showed positive impacts on AmB yield in flask fermentation, which is 22.8% to 26.4% higher than that of the original mutant *S. nodosus*. The production of AmB in *metH* overexpression strain reached 5.03 g/L within 120 h fermentation time, about 26.4% higher than that of mutant *S. nodosus*, which needed 144 h fermentation time for almost the same AmB production titer. Our results indicated the application of gene overexpression strategy based on the comparative metabolomics analysis would be very effective for the rational design of other antibiotics production improvement.

## Methods

### Bacterial strain, medium, and culture conditions

*S. nodosus* ZJB2016050 (CCTCC M2017426, China Center for Type Culture Collection, Wuhan, China) used throughout this study was isolated after N-methyl-N-nitroso-N-nitroguanidine (NTG) and ultraviolet (UV) mutagenesis of wild-type *S. nodosus*. The detailed ultraviolet mutagenesis method was reported by Zhang and co-workers [23]. All strains and recombinant plasmids with characteristics and resources used in this article were listed in Additional file 1: Table S1.

The composition of the fermentation medium contained 69 g/L glucose, 25 g/L beef extract, 9 g/L CaCO_3_ and 0.1 g/L KH_2_PO_4_. The composition of the seed medium contained 15 g/L tryptone, 10 g/L yeast extract, 10 g/L glucose, 5 g/L NaCl and 1 g/L CaCO_3_. Luria–Bertani (LB) medium and GYM medium were used for *E. coli* and *S. nodosus* cultivation, respectively. The composition of the LB medium contained 10 g/L tryptone, 5 g/L NaCl, 5 g/L yeast extract (additional 20 g/L agar for solid medium). The composition of the GYM medium contained 4 g/L glucose, 4 g/L yeast extract, 10 g/L malt extract, 2 g/L CaCO_3_ and 20 g/L agar. The composition of the MS medium contained 20 g/L soybean meal, 20 g/L mannitol and 20 g/L agar. Before autoclaving, the pH value of the fermentation, seed and LB medium was adjusted to 7.0, and the pH value of the GYM and MS medium was adjusted to 7.2. The agar medium was prepared by adding soybean meal into a certain volume of distilled water and boiling for 1 h, then the volume was filled up to 1 L by distilled water. Finally, mannitol and agar were quantitatively added into the medium and autoclaved. All used primers are listed in Additional file 1: Table S2.

The strain was cultivated on MS agar plate at 26 °C for 4–6 days to isolate a single colony. The isolated pure culture was then grown on the GYM medium for 7 days for the harvest of spores. The 1 mL resulting spore suspension was then transferred into a 50 mL seed medium in a 250-mL shake flask and incubated for 48 h at 26 °C. Seed cultures (2 mL) were then inoculated into 50 mL fermentation medium in a 500-mL shake flask and incubated at 26 °C for 168 h.

### Analytical methods

AmB concentration was measured by high-performance liquid chromatography (HPLC) (3200; LDC ANALYTICAL INC., New York, USA) equipped with a Agilent C18 reversed-phase column (5 μm, 4.6 × 150 mm, Agilent Technologies Inc., USA) and a UV–vis detector. Culture samples (0.15 mL) mixed with the nine times volume DMSO was shaken intermittently in a rotary shaker at 50 °C for 30 min. After centrifugation at 12,000*g* for 2 min, the supernatant filtered through a 0.22 μm organic filter was subjected to HPLC analysis. The mobile phase was comprised of water/acetonitrile/methanol (9:7:4, v/v/v) with a flow rate of 1.0 mL/min. the pH value of the mobile phase was adjusted to 5.0 with glacial acetic acid. The column temperature was 25 °C and UV detector was set at 304 nm (0–12 min) and 405 nm (12–26 min). The AmB standard sample was purchased from Sigma-Aldrich (CAS: 1397-89-3).

The dry cell weight (DCW) of strains were measured as follows. In detail, 1 mL of culture was sampled and centrifuged at 12,000*g* for 10 min. To remove calcium carbonate, appropriate amount of acetic acid was added and reacted for 10 min. The mycelium was washed twice with sterilized water, centrifuged at 12,000*g* for 10 min, and dried to constant weight at 80 °C. The residual sugar in the fermentation broth was quantified using a commercial glucose assay kit. The pH value was detected using a commercial pH meter.

### Sample preparation for metabolic analysis

According to the AmB production curve (Fig. 1), samples for metabolic analysis were harvested at 24 h when the AmB accumulation began to be divergent (> 0.5 g/L) between mutant *S. nodosus* and wild-type *S. nodosus.* Samples were taken at 24, 72, 120 and 156 h, respectively.

For metabolites extraction, 2 mL cultures were sampled and centrifuged below 4 °C at 12,000*g* for 5 min. the supernatant was collected and washed three times with 1.5 mL cold saline, centrifuged again below 4 °C at 12,000*g* for 5 min. The supernatant was collected and froze rapidly in liquid nitrogen, then stored at − 80 °C. Before testing, the samples were placed at − 20 °C for 30 min, and then thawed in an ice bath for 4 min. Another 0.8 mL cold methanol/water (1:1, v/v) and two steel beads were added into the cell pellet.

Cell samples were placed in the TissueLyser at 35 Hz and grind for 4 min. After grinding, the samples were precipitated at − 20 °C for two hours, and centrifuged at 12,000*g* for 20 min at 4 °C. The supernatant was then taken out for liquid chromatography-mass spectrometry (LC–MS) analysis.

### LC–MS analysis

LC–MS was performed to analyze the metabolite samples using a Waters 2777C UPLC system (Waters, Milford, USA) equipped with an ACQUITY UPLC BEH C18 spectrum column (100 mm × 2.1 mm, 1.7 μm, Waters, Milford, USA). The spectrum column temperature was 50 °C and flow rate was set at 0.4 mL/min. The metabolites were eluted with the following gradient: 0–2 min, 100% mobile phase A; 2–11 min, 0–100% mobile phase B; 11–13 min, 100% mobile phase B; 13–15 min 0–100% mobile phase A. mobile phase A is deionized water with 0.1% formic acid, and the mobile phase B is methanol with 0.1% formic acid.

The mass spectrometer model was high-resolution tandem mass spectrometry Xevo G2-XS QTOF (Waters, Milford, USA). Small molecules washed from liquid chromatography columns were collected using the positive ion mode and negative ion mode modes. The capillary voltage and conical hole voltage in two positive and negative ion modes are 3 kV, 40 V and 1 kV, 40 V., respectively centroid data were collected using full-information tandem mode, the molecular size of primary mass spectrometry scanning was 50–1200 Da, scanning time was 0.2 s. Then break all the parent ions with 20–40 eV set energy, and the LE signal correction is performed every 3 s for all the collected fragments scanning time 0.2. Normally, to assess the accuracy and stability of the equipment during detection and collection process, QC samples (mixture of all samples) were prepared in advance and tested every 10 samples. Perform data alignment and normalization for the complete data set, composed of multiple analytical blocks [25].

The LC–MS raw data were analyzed by Progenesis QI (Waters Corporation, Milford, USA) software using the following parameters [26]. The parameters used were retention time range 0.5–14.0 min, mass range 50–1000 Da, mass tolerance 0.01 Da. Isotopic peaks were excluded for analysis, noise elimination level was set at 10.00, minimum intensity was set to 15% of base peak intensity, and finally, RT tolerance was set at 0.01 min. The Excel file was obtained with three-dimension data sets including m/z, peak RT and peak intensities, and RT-m/z pairs were used as the identifier for each ion. The resulting matrix was further reduced by removing any peaks with missing value in more than 60% samples. The internal standard was used for data QC. The positive and negative data were combined to get a combine data set which was imported into SIMCA-P+ 14.0 software package (Umetrics, Umeå, Sweden) [27]. And then the data was processed by two different standardized methods such as centralization (mean-centering) and automatic specification (unit variance scaling, UV).

LC/MS raw data uses Progenesis QI software (Waters Corporation, Milford, USA) for baseline filtering, peak identification, integration, peak alignment, retention time correction, standardization and normalization, and finally a retention time, mass-to-charge ratio and peak are obtained Intensity data matrix. 7758 metabolites were identified under the scoring standard of Progenesis QI software. Use the *p* value obtained by univariate analysis and the VIP value obtained by multivariate analysis to screen for differential metabolites. The screening conditions are *p* value < 0.05 (T-test) and VIP > 1 (Simca-p 14.1), which satisfy both the metabolites of the condition are the differential metabolites. The selected differential metabolites and their corresponding genes are integrated into the data and used as the raw data of the MetaboAnalyst software (https://www.metaboanalyst.ca). Enter the MetaboAnalyst software homepage, select the “Joint pathway Analysis” module, upload the data, enter the above processed gene list and the corresponding metabolite list data, perform ID mapping on the designated gene and metabolite identifier, and then perform the gene with the KEGG database map with metabolites to get the result table, click submit to enter the parameter analysis, and get the pathway enrichment analysis result. It is similar to the metabolic pathway analysis result figure (Additional file 1: Figure S1). We plotted the top 10 metabolic pathways with the lowest *p* value (Additional file 1: Figure S2).

### Chemical reagent

Unless otherwise noted, all the reagents and solvents used in this study were purchased from Sigma Chemical Company (St. Louis, MO, USA) and Sangon Biotech (Shanghai, China) at the analytical grade.

### Data analysis

All the experiments were carried out for at least five replicates, and the values were shown as mean ± standard deviations. The *p* value was performed with t tests and fold change analysis to analyze the data obtained from LC–MS. The levels of metabolites in mutant *S. nodosus* and wild-type *S. nodosus* were considered significantly different at *p* value < 0.05.

Principle component analysis and (orthogonal) partial least-squares-discriminant analysis were carried out to visualize the metabolic alterations among experimental groups, after mean centering and unit variance scaling. Variable importance in the projection ranks the overall contribution of each variable to the (O)PLS-DA model, and those variables with VIP > 1 are considered relevant for group discrimination. In this study, in order to guard against overfitting, the default 7-round cross-validation was applied with a seventh of the samples being excluded from the mathematical model in each round.

## Supplementary Information

**Additional file 1: Table S1.** Strains and plasmids used in this study. **Table S2.** Primers used in this study. **Table S3.** Annotated metabolites detected by LC–MS/MS in *S. nodosus* ZJB2016050 and *S. nodosus* ZJB20140315 among different groups and related pathways. **Figure S1.** Metabolic pathway analysis revealing metabolic impacts of metabolic pathway to the key fermentation of *S. nodosus* via comparison. **Figure S2.** The enrichment of metabolic pathway. **Figure S3.** The production of AmB for different metabolites addition with strain *S. nodosus* ZJB2016050. **Figure S4.** RT-qPCR analysis of transcriptional levels of single gene expression in *S. nodosus* ZJB2016050.

## Data Availability

Not applicable.

## Abbreviations

AmB: Amphotericin B
AmA: Amphotericin A
DCW: Dry cell weight
HPLC: High performance liquid chromatography
PCA: Principal component analysis
(O)PLS-DA: (Orthogonal) partial least square discriminate analysis
VIP: Variable importance in projection
QC: Quality control
