# Supplementary material for: Comparative metabolomics analysis of amphotericin B high-yield mechanism for metabolic engineering
Source: Microb Cell Fact. 2021 Mar 9;20:66. doi: 10.1186/s12934-021-01552-z (PMC7945361; doi:10.1186/s12934-021-01552-z)
Supplement: Supplementary file 1 — Additional file 1: Table S1. Strains and plasmids used in this study. Table S2. Primers used in this study. Table S3. Annotated metabolites detected by LC–MS/MS in S. nodosus ZJB2016050 and S. nodosus ZJB20140315 among different groups and related pathways. Figure S1. Metabolic pathway analysis revealing metabolic impacts of metabolic pathway to the key fermentation of S. nodosus via comparison. Figure S2. The enrichment of metabolic pathway. Figure S3. The production of AmB for different metabolites addition with strain S. nodosus ZJB2016050. Figure S4. RT-qPCR analysis of transcriptional levels of single gene expression in S. nodosus ZJB2016050. [file 12934_2021_1552_MOESM1_ESM.docx]

**Additional file**

**Comparative** **metabolomics analysis of amphotericin B high-yield mechanism for metabolic engineering**

Bo Zhang^1,2^, Yu Chen^1, 2^, Sheng-Xian Jiang^1,2^, Xue Cai^1, 2^, Kai Huang^1,2^, Zhi-Qiang Liu^1,2*^ and Yu-Guo Zheng^1,2^

^1^*Key Laboratory of Bioorganic Synthesis of Zhejiang Province, College of Biotechnology and Bioengineering, Zhejiang University of Technology, Hangzhou 310014, P. R. China*

^2^*Engineering Research Center of Bioconversion and Bio-purification, Ministry of Education, Zhejiang University of Technology, Hangzhou 310014, P. R. China*

***Correspondence:** Professor Zhi-Qiang Liu, Department, Key Laboratory of Bioorganic Synthesis of Zhejiang Province, College of Biotechnology and Bioengineering, Zhejiang University of Technology, Hangzhou 310014, China

Tel: +86-571-88320614, Fax: +86-571-88320630, E-mail: [microliu@zjut.edu.cn](mailto:microliu@zjut.edu.cn)**Table S1 Strains and plasmids used in this study**

| Strains or plasmids | characteristics | Reference or source |
| --- | --- | --- |
| ***S. nodosus*** |  |  |
| ZJB2016050 | Mutant strain from *Streptomyces* sp. ZJB20140315 amphotericin producer | Zhang et al., 2018  Strain number: CCTCC NO: M2017426 |
| ZJB20140315 | wild-type strain amphotericin producer | Zhang et al., 2018 |
| pJTU1278 | ZJB2016050 with pJTU1278 vector | This work |
| purN | ZJB2016050 with pJTU-EpurN to overexpress *purN* | This work |
| metH | ZJB2016050 with pJTU-EmetH to overexpress *metH* | This work |
| glyA | ZJB2016050 with pJTU-EglyA to overexpress *glyA* | This work |
| metF | ZJB2016050 with pJTU-EmetF to overexpress *metF* | This work |
| purH | ZJB2016050 with pJTU-EpurH to overexpress *purH* | This work |
| fmt | ZJB2016050 with pJTU-Efmt to overexpress *fmt* | This work |
| ***E.coli*** |  |  |
| DH5α | General cloning host | Vazyme Biotech Co.,Ltd |
| ET12567 (pUZ8002) | Strain for intergeneric conjugation |  |
| **Recombinant plasmids** | |  |
| pJTU1278 | *E. coli-Streptomyces* conjugative vector |  |
| pJTU-EpurN | pJTU1278 vector with ermE*p promoter and *purN* gene, phosphoribosylglycinamide formyltransferase gene | This work |
| pJTU-EmetH | pJTU1278 vector with ermE*p promoter and *metH* gene, methionine synthase gene | This work |
| pJTU-EglyA | pJTU1278 vector with ermE*p promoter and *glyA* gene, serine hydroxymethyltransferase gene | This work |
| pJTU-EmetF | pJTU1278 vector with ermE*p promoter and *metF* gene, methylenetetrahydrofolate reductase gene | This work |
| pJTU-EpurH | pJTU1278 vector with ermE*p promoter and *purH* gene, bifunctional phosphoribosylaminoimidazolecarboxamide  formyltransferase gene | This work |
| pJTU-Efmt | pJTU1278 vector with ermE*p promoter and *fmt* gene, methionyl-tRNA formyltransferase gene | This work |

**Table S2 Primers used in this study**

| **Primer** | **Sequence（5’→3’）** | **Source** |
| --- | --- | --- |
| ***Recombinant plasmid construction*** | | |
| GlyA-F | GGAGGACCCCAAAGCTTATGTCGCTTCTGAACACACC | This study |
| GlyA-R | GGGCGAATTGGGTACCTTACTTCCCGAGCCCCGGGTA | This study |
| *PurN*-F | CACTCCACAGGAGGACCCCAAAGCTTGTGGCCGAGAC | This study |
| *PurN*-R | GGGCGAATTGGGTACCTCACTGGATTACTACC | This study |
| *Fmt*-F | GAGGACCCCAAAGCTTATGAGGCTCGTCTTCGCAGGTA | This study |
| *Fmt*-R | GCGAATTGGGTACCTCAGCTCCCGAGGCTCTC | This study |
| *MetH*-F | GAGGACCCCAAAGCTTATGGCCTCGTCGCCACA | This study |
| *MetH*-R | GGCGAATTGGGTACCTCAGCGGGCGTTGAAGTAC | This study |
| *MetF*-F | GAGGACCCCAAAGCTTATGGCCCTCGGAACC | This study |
| *MetF*-R | GGGCGAATTGGGTACCCTAGGTCCGCTGAGAATG | This study |

**Table S3 Annotated metabolites detected by LC-MS/MS in *S. nodosus* ZJB2016050 and *S. nodosus* ZJB20140315 among different groups and related pathways**

| NO | metabolites | Rt(min) | m/z | Formula | VIP | *S. nodosus* ZJB2016050 | | | *S. nodosus* ZJB20140315 | | | pathway |
| --- | --- | --- | --- | --- | --- | --- | --- | --- | --- | --- | --- | --- |
|  |  |  |  |  |  | 24 h to 72 h | 72 h to 120 h | 120 h to 156 h | 24 h to 72 h | 72 h to 120 h | 120 h to 156 h |  |
| 1 | Glutathione ^24*, 72*, 120*^ | 6.88 | 327.1527 | C_10_H_17_N_3_O_6_S | 1.13307 | ↑ | ↓ | ↓ | ↓ | ↑ | ↓ | Amino acid metabolism |
| 2 | Ergothioneine ^24*, 72*, 120*^ | 0.80 | 183.1135 | C_9_H_11_NO_2_ | 1.21798 | ↑ | ↓ | ↑ | ↑ | ↓ | ↑ | Amino acid metabolism |
| 3 | Histidinol ^24*, 72*^ | 0.66 | 116.0717 | C_5_H_9_NO_2_ | 1.27166 | ↓ | ↓ | - | ↓ | ↑ | ↓ | Amino acid metabolism |
| 4 | Methionine ^24*, 72*, 120*,156*^ | 4.38 | 150.0591 | C_5_H_11_NO_2_S | 1.20266 | - | ↑ | ↑ | ↑ | ↓ | ↑ | Amino acid metabolism |
| 5 | Methylhistamine ^24*, 72*, 120*,156*^ | 7.15 | 970.5352 | C_6_H_13_NO_2_ | 1.79056 | - | - | ↑ | ↑ | ↓ | ↑ | Amino acid metabolism |
| 6 | SAM ^24*, 156*^ | 9.57 | 399.1472 | C_15_H_22_N_6_O_5_S | 1.73878 | ↓ | ↓ | ↓ | - | ↓ | ↑ | Amino acid metabolism |
| 7 | Serine ^24*, 72*^ | 3.49 | 106.0508 | C_3_H_7_NO_3_ | 1.15362 | ↓ | ↑ | ↓ | ↓ | ↑ | ↓ | Amino acid metabolism |
| 8 | Tetrahydrodipicolinate ^24*, 72*, 120*,156*^ | 0.69 | 132.0663 | C_5_H_9_NO_3_ | 1.85378 | ↓ | ↓ | - | ↑ | ↓ | - | Amino acid metabolism |
| 9 | Lactose ^24*, 72*, 120*^ | 1.50 | 342.1162 | C_12_H_22_O_11_ | 1.1633 | - | ↑ | ↑ | ↑ | ↑ | ↓ | central carbon metabolism |
| 10 | Sedoheptulose-7-phosphate ^24*, 120*^ | 0.59 | 289.1634 | C_7_H_15_O_10_P | 1.3424 | ↓ | ↑ | ↓ | - | - | - | central carbon metabolism |
| 11 | 5,10-Methylene-THF ^24*^ | 4.63 | 440.1687 | C_20_H_23_N_7_O_6_ | 1.20303 | ↓ | ↑ | ↓ | ↑ | ↓ | ↓ | Folate biosynthesis and one carbon pool by folate |
| 12 | 5,10-Methyl-THF ^24*, 72*, 120*^ | 7.83 | 457.1712 | C_20_H_22_N_7_O_6_ | 1.18003 | ↓ | ↑ | ↓ | ↓ | ↑ | ↓ | Folate biosynthesis and one carbon pool by folate |
| 13 | Tetrahydrofolic acid ^24*, 72*, 120*,156*^ | 4.27 | 463.2058 | C_19_H_23_N_7_O_6_ | 1.38502 | ↑ | ↓ | ↓ | - | - | - | Folate biosynthesis and one carbon pool by folate |
| 14 | 5-Aminoimidazole-4-carboxamide ^24*, 72*, 120*^ | 3.26 | 329.0076 | C_4_H_6_N_4_O | 1.43820 | ↑ | ↓ | ↑ | ↑ | ↓ | - | Purine and pyrimidine metabolism |
| 15 | 5'-phosphoribosyl-N-formylglycinamide(FGAR)^24*^ | 11.91 | 281.0521 | C_8_H_15_N_2_O_9_P | 1.34232 | - | ↓ | - | - | ↑ | ↑ | Purine and pyrimidine metabolism |
| 16 | Adenine ^24*^ | 3.46 | 136.0628 | C_5_H_5_N_5_ | 1.27433 | ↑ | ↑ | ↓ | - | - | ↓ | Purine and pyrimidine metabolism |
| 17 | Inosine ^24*, 72*, 120*,156*^ | 3.40 | 251.0784 | C_10_H_12_N_4_O_5_ | 1.35515 | ↑ | ↓ | - | - | - | - | Purine and pyrimidine metabolism |
| 18 | Heptadecanoic acid(TAG-C17:0) ^24*, 72*, 120*^ | 10.61 | 504.3246 | C_17_H_34_O_2_ | 1.73992 | ↓ | ↓ | ↑ | ↓ | ↑ | ↑ | Fatty acid metabolism |
| 19 | Isomyristic acid(TAG-iC14:0) ^24*, 72*, 120*^ | 12.17 | 211.2059 | C_14_H_28_O_2_ | 1.26533 | ↓ | ↓ | ↑ | - | - | ↑ | Fatty acid metabolism |
| 20 | Lysophosphatidylcholine ^24*, 72*, 120*,156*^ | 9.65 | 496.4223 | C_24_h_50_NO_7_P | 2.64384 | ↓ | ↓ | - | - | - | ↑ | Fatty acid metabolism |
| 21 | phosphatidic acid ^24*, 72*, 120*^ | 9.10 | 435.2520 | C_13_H_12_O_9_ | 1.15353 | ↓ | ↑ | ↓ | - | - | - | Fatty acid metabolism |
| 22 | TG(10:0/8:0/8:0) ^24*, 72*, 120*^ | 11.20 | 425.3657 | C_26_H_58_O_6_ | 1.14155 | ↓ | ↓ | ↑ | - | - | ↑ | Fatty acid metabolism |
| 23 | TG(19:0/22:0/10:0) ^24*, 72*, 120*^ | 12.04 | 788.5888 | C_51_H_108_O_6_ | 2.18172 | ↓ | ↓ | ↑ | ↑ | - | ↑ | Fatty acid metabolism |
| 24 | TG(8:0/12:0/10:0) ^24*, 72*^ | 11.68 | 548.4392 | C_30_H_66_O_6_ | 1.55774 | ↓ | ↓ | ↑ | ↑ | ↑ | ↑ | Fatty acid metabolism |
| 25 | TG(8:0/i-24:0/i-15:0) ^24*, 72*, 120*^ | 12.32 | 624.2369 | C_47_H_100_O_6_ | 1.67754 | ↓ | ↓ | ↑ | - | - | ↑ | Fatty acid metabolism |
| 26 | TG(i-24:0/8:0/14:0) ^24*, 72*^ | 12.11 | 768.7095 | C_46_H_98_O_6_ | 2.99051 | ↓ | ↓ | ↓ | - | - | ↑ | Fatty acid metabolism |

24*, 72*, 120* and 156* represented the significant difference of metabolites at 24, 72, 120 and 156h, respectively, in *S. nodosus* ZJB2016050 relative to that in *S. nodosus* ZJB20140315. “↑” indicated up, “↓” indicated down, “-” indicated constan


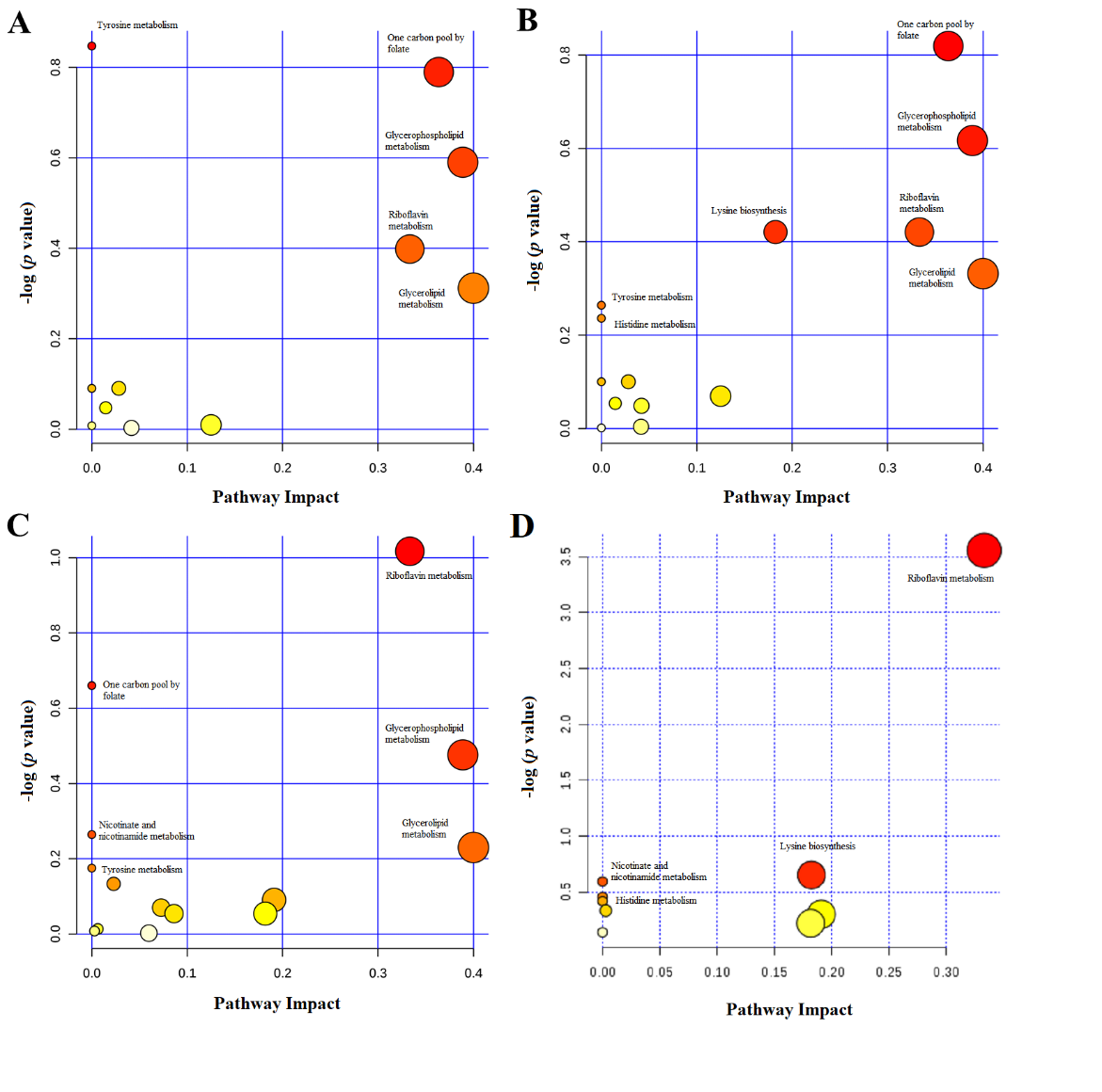


**Figure S1 Metabolic pathway analysis revealing metabolic impacts of metabolic pathway to the key fermentation of *S. nodosus* via comparison**

A-D: The pathway overview of *S. nodosus* ZJB2016050 and *S. nodosus* ZJB20140315 at 24 h, 72 h, 120 h and 156 h. The pathway impact is calculated as the sum of the importance measures of the matched metabolites normalized by the sum of the importance measures of all metabolites in each pathway. The node color is based on its *p* value and the node radius is determined based on their pathway impact values. At 24 h, one carbon pool by folate metabolism, tyrosine metabolism, glycerophospholipid metabolism, riboflavin metabolism and glycerolipid metabolism were more significant; at 72 h, one carbon pool by folate metabolism, glycerophospholipid metabolism, lysine biosynthesis, riboflavin metabolism, tyrosine metabolism and histidine metabolism were more significant; at 120 h, riboflavin metabolism, one carbon pool by folate metabolism, glycerophospholipid metabolism, glycerolipid metabolism, nicotinate and nicotinamide metabolism were more significant; at 156 h, riboflavin metabolism, lysine synthesis, nicotinate and nicotinamide metabolism were more significant.

**Figure S2 The enrichment of metabolic pathway**

Mapping the information of different metabolites to the KEGG database could help obtain the metabolic pathway enrichment with different metabolites listed and different types of statistical figure. *P* value indicated that the differential metabolites were enrichment in this metabolic pathway. When the *p* value was less than 0.05, the function was generally considered credible, and the p value test had been passed the Holm’s Sequential Bonferroni Procedure (Holm method) correction calculation and the False Discovery Rate (FDR) correction calculation.

The bar graph was used to visualize the KEGG enrichment results. The first 10 metabolic pathways with the lowest *p* value were plotted. The X-axis shows the different metabolisms. The Y-axis shows the negative log-base e of *p* value. The larger -log (*p* value) value was, the higher the significance of the pathway was.


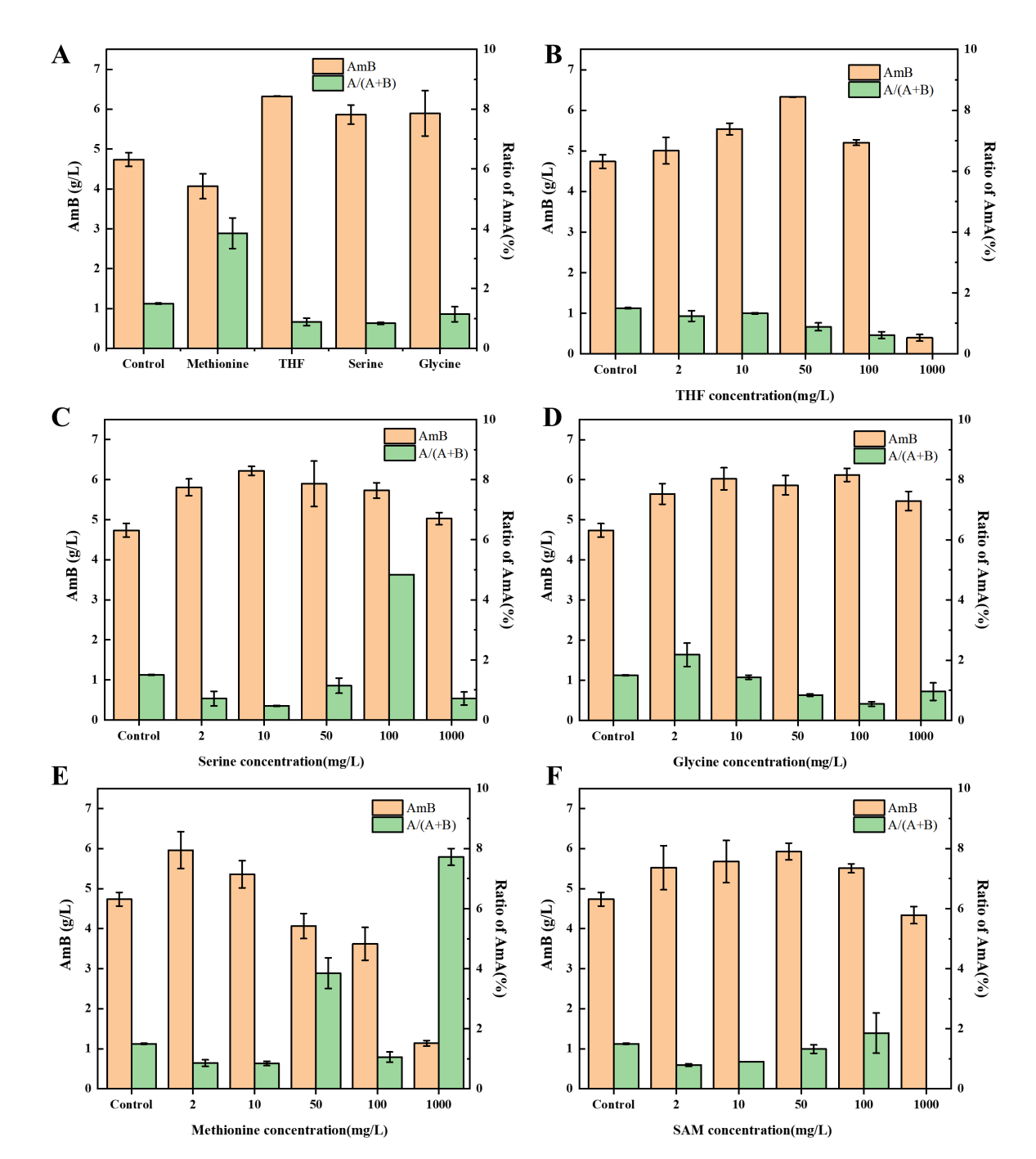


**Figure S3 The production of AmB for different metabolites addition with strain *S. nodosus* ZJB2016050.**

(A) Effects of amino acids addition (50 mg/L) on AmB production. (B) Effects of different THF concentration on AmB production. (C) Effects of different serine concentration on AmB production. (D) Effects of different glycine concentration on AmB production. (E) Effects of different methionine concentration on AmB production. (F) Effects of different SAM concentration on AmB production. Error bars show standard derivation among three experiments.


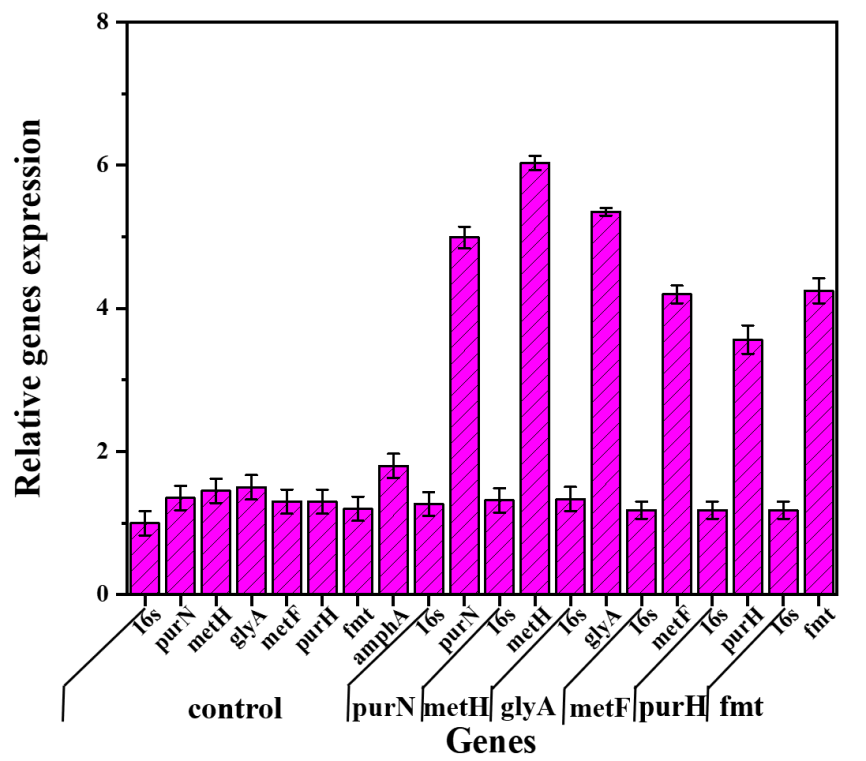


**Figure S4 RT-qPCR analysis of transcriptional levels of single gene expression in *S. nodosus* ZJB2016050.**

All experiments were performed in triplicate; error bars denote standard deviation of the mean.
